# Research on the impact of the development of green finance in the China region on residents’ health

**DOI:** 10.3389/fpubh.2023.1250600

**Published:** 2023-08-10

**Authors:** Shuhao Fan, Fanchao Kong, Cheng Li

**Affiliations:** ^1^Quality Management Department, Shandong Cancer Hospital and Institute, Shandong First Medical University and Shandong Academy of Medical Sciences, Jinan, China; ^2^School of Statistics and Mathematics, Shandong University of Finance and Economics, Jinan, China

**Keywords:** green finance, residents’ health, health care accessibility, carbon intensity, non-linear characteristics

## Abstract

In the context of implementing the strategy of “double carbon” and “healthy China,” this paper firstly measures the level of green finance development and the comprehensive index of health care accessibility in each province by using the entropy weight method based on 30 provincial panel data from 2007 to 2021. A panel fixed effects model was also used to empirically analyze the effect of regional green finance development on the improvement of residents’ health. In addition, a panel threshold model was constructed to empirically test the threshold effect of green finance on residents’ health under the influence of four external environments: carbon intensity level, healthcare accessibility, residents’ living standard and human capital level. The empirical results show that the regional green financial development in China significantly improves the health level of residents. And the impact has significant regional heterogeneity, as shown in the improvement effect is more significant for the provinces in the central and western regions. In addition, the impact of green financial development on the health level of residents in China is non-linearly influenced by external environmental factors. The improvement effect of green finance on residents’ health level is more significant in the provinces with higher carbon intensity level, residents’ living standard, human capital level and lower accessibility to medical services. In this regard, regional governments should continue to build and optimize a synergistic development ecosystem of green finance and public health, give full play to the advantages of financial leverage, promote green, low-carbon and high-quality economic and social development, and realize the beautiful vision of harmonious coexistence between human beings and nature.

## 1. Introduction

The 20th National Congress of the Communist Party of China delivered a significant strategic plan in its report, emphasizing the “promotion of green development and the harmonious coexistence of humans and nature.” It is emphasized that we should firmly establish and practice the concept of green water and green mountains is the silver mountain of gold, improve the financial policy and standard system to support green development, and make efforts to promote green and low-carbon economic and social development. The development of green finance is an inevitable requirement for promoting green development (1). Green finance is a catalyst and gas pedal to promote green and low-carbon development, and is a financial activity that addresses climate change, supports environmental improvement and promotes efficient use and conservation of resources. It can actively guide the flow of financial resources to the green economy by attracting investors to invest in environmental protection and low-carbon technology projects, promoting green economic development and environmental improvement, and achieving effective improvement in the quality of economic development and reasonable growth in quantity (2). It is this power to promote a win–win situation for both the economy and the environment that makes green finance not only play a positive role in promoting economic development, but also play an important role in major issues that concern everyone’s health. The report of The 20th National Congress of the Communist Party of China has made “healthy China” another important aspect of China’s overall development goal for 2035, proposing to give priority to people’s health, improve people’s health promotion policies, promote the construction of a healthy China, and better protect people’s health.

With the improvement of economic society and people’s living standard, people pay more and more attention to the quality of life and health safety, and health needs show diversified and differentiated characteristics. To meet the growing health needs of the people, we need to explore new paths. At a time when we are facing increasingly serious environmental problems, the flourishing development of green finance can provide important support for the demand for funds and optimal allocation of resources in related fields. This undoubtedly provides an important opportunity to effectively control and reduce the impact of environmental pollution on public health (3). However, although the potential of green finance in promoting environmental protection and sustainable development has been widely recognized, there is a relative lack of research on its impact on public health. In this context, an in-depth study of the effect of regional green finance development in China on the residents’ health and its mechanism of action can provide some reference basis for the government to formulate green finance policies, promote the green development of the economy and the construction of a healthy China, and realize the national strategy of coordinated development of the health of the population and the economy and society.

## 2. Literature review and research hypothesis

### 2.1. Literature review

A good natural environment is the material basis for human survival and development. The environment provides humans with the necessary living and production sites, but environmental factors are also inseparable from human health. Studies by Dockery et al. (4) and Brook et al. (5) have shown that emissions of airborne particulate matter, sulfur dioxide, nitrogen dioxide, and other pollutants pose a threat to human health, producing various health problems such as heart and lung disease, respiratory disease, and cancer. Moreover, environmental pollution not only makes individuals suffer from diseases, but also imposes a heavy burden on socio-economic development, including the increase of health care costs and the decrease of labor productivity (6). However, the traditional financial mechanism is inadequate in the allocation of funds, often favoring those projects that can obtain significant returns in the short term, while ignoring the need for long-term investment, but has a profound impact on society and the environment, such as clean energy projects, sewage treatment projects and other types of environmental protection projects (7). The environmental protection projects with the popularity of environmental awareness and the severity of global climate change, green finance, as a kind of commitment to improve environmental quality, is increasingly seen by scholars as an important driver of green development. A study by Weber and Feltmate (8) points out that green finance can attract and direct capital to environmental projects by providing low-interest loans and issuing green bonds, thus improve environmental quality. Taghizadeh-Hesary and Yoshino (9) states that green finance is essential for financing renewable and green energy projects, which can reduce the negative health impacts of carbon emissions, develop climate-resilient infrastructure for cities, and ensure environmental sustainability. Governments have sought to address adverse environmental health impacts by adopting sustainable energy sources and, more broadly, by using green finance to invest in sustainable project development (10). Xu and Zhu (11) show that green finance policies and efficient green governance play a key role in achieving sustainable development and improving public health, and can significantly reduce environmental pollution and carbon emission levels.

In summary, most of the existing studies focus on the direct impact of green finance on environmental protection, while there are relatively few quantitative studies on the impact of green finance on the health of the population. This change in perspective will help us to comprehensively understand the social benefits of green finance. In addition, the mechanism of the effect of green finance on residents’ health has not been strongly tested. This paper uses a threshold effect model to further reveal the nonlinear characteristics of the effect of green finance on residents’ health improvement under the moderating influence of external environmental factors such as carbon intensity level, healthcare accessibility, residents’ living standard and human capital level.

### 2.2. Research hypothesis

Green finance, also known as ecological or sustainable finance, regulates and guides the flow of financial capital. It encourages consumers and financiers to focus on green and environmental sectors. Its purpose is to optimize resource allocation, combat climate change, protect the ecological environment, promote regional green and low-carbon transformations, and facilitate harmonious economic, social, and natural development (8). The impact of green finance on residents’ health is primarily reflected in several aspects: Firstly, green finance supports clean energy, energy conservation, emission reduction, and pollution treatment projects, which reduce greenhouse gas and air pollutant emissions, improving air quality and reducing the risk of respiratory and cardiovascular diseases among residents. Secondly, green finance can bolster initiatives such as ecological restoration, forest protection, and biodiversity conservation, enhancing the service functions of ecosystems. These projects provide ecological benefits such as clean water, soil conservation, and disaster prevention, which help ensure residents’ drinking water safety, food security, and livelihood stability. Finally, green finance can foster sustainable projects such as green buildings, green transportation, and green consumption. These initiatives improve the sustainability of cities and communities, optimize residents’ living environments and travel patterns, and promote residents’ physical and mental health and well-being (12). Based on the above analysis, this paper proposes the following hypotheses:

*H1*: Green finance significantly promotes the improvement of residents' health levels.

Green finance, with its advantages of environmental protection, efficiency and sustainability, helps more regions and residents to enjoy the benefits of environmental protection. But its impact on residents’ health level may be influenced by the threshold effect of external environmental factors such as the level of carbon intensity, accessibility of medical services, residents’ living standard and human capital level in each region to regulate, triggering the dynamic evolution of residents’ health improvement effect.

First, the effect of green finance on the health of the population depends, to some extent, on the environmental conditions of each region, i.e., the level of carbon intensity. Regions with high carbon intensity levels usually face more serious environmental problems, and this environmental pressure provides a greater market demand and opportunity for green finance. As a financial innovation tool, green finance can effectively provide financial support to guide and motivate enterprises to invest in environmental protection and promote the development of new technologies, products, and business models, thus helping to solve environmental problems, reduce regional carbon emissions, and improve environmental quality, thus enhancing the health of residents (13). Second, the effect of green finance on residents’ health depends to some extent on the health care resources, i.e., the accessibility of health care services, in each region. In areas with low accessibility to healthcare services, residents may face greater health challenges because they may not have access to sufficient healthcare resources to cope with health problems caused by environmental pollution. At this point, environmental improvements from green finance may have a more significant positive impact on the health of the population (14, 15). Third, the effect of green finance on the health of the population depends to some extent on the standard of living of the population in each region. In regions with lower living standards, people may be more concerned with basic living needs and have relatively lower demand for environmental protection projects and technologies, so they naturally cannot make full use of green finance products and services, which weakens its positive impact on residents’ health. In contrast, in areas with higher living standards, the higher awareness of environmental protection and the demand for environmental projects are more likely to direct green financial investments to environmental industries, thus positively affecting the health of the population (16, 17). Fourth, the effect of green finance on the health of the population depends to some extent on the internal absorptive capacity of each region, i.e., the level of human capital. Green finance, as the integration of a new generation of financial models and environmental protection concepts, has a higher threshold of practice compared to the general financial industry, and if personnel do not have sufficient financial and environmental knowledge, they will naturally be unable to fully understand and utilize green financial products and services, which will limit the ability of green finance in improving the health of the population (18). At the same time, the level of human capital is also an important factor in driving health improvement. Because individuals with higher levels of education are better able to access, understand, and apply health-related information and knowledge, and are better able to understand and adopt behaviors to prevent disease and maintain health (19). Based on this, this paper proposes the following research hypothesis:

*H2*: The contribution of green finance to the residents’ health is more significant in the provinces with higher carbon intensity levels.

*H3*: The contribution of green finance to the residents' health is more significant in provinces with lower accessibility to healthcare services.

*H4*: In provinces with a higher standard of living, the contribution of green finance to the residents' health is more significant.

*H5*: The contribution of green finance to the residents’ health is more significant in provinces with higher levels of human capital.

## 3. Empirical models and variables

### 3.1. Econometric model

In order to explore the impact of China’s regional green finance development level on residents’ health, this paper constructs the basic measurement model as follows:


(1) $${\mathrm{Health}}_{it}=\beta_{0}+\beta_{1}{\mathrm{Gfi}}_{it}+\rho {\mathrm{Control}}_{it}+\lambda_{i}+\eta_{t}+\varepsilon_{it}$$

where ${\mathrm{Health}}_{it}$ represents the health level of residents in province *i* in period *t*, and ${\mathrm{Gfi}}_{it}$ represents the green financial development index of province *i* in period *t*, and specifically includes four subdivisions of green credit, green investment, green insurance and green securities. ${\mathrm{Control}}_{\mathrm{it}}$ denotes a series of control variables, $\lambda_{i}$ are individual fixed effects, $\eta_{t}$ are time fixed effects, and $\varepsilon_{it}$ is the random disturbance term. $\beta_{0}$ denotes the model intercept term, $\beta_{1}$ are the coefficients of green finance variables, and the magnitude and direction of the coefficients reflect their effects on residents’ health.

In addition, the impact of regional green financial development level on residents’ health in China may be subject to a threshold effect moderated by the external environment of each region. To further reveal whether there are nonlinear characteristics arising from green financial development in the level of residents’ health, this paper uses a panel threshold model to test the nonlinear mechanism. The model is constructed as follows:


(2) $$\begin{array}{l} {\mathrm{Health}}_{it}=\beta_{0}+\beta_{1}{\mathrm{Gfi}}_{it}\cdot I\left(T_{it}\leq \gamma \right)+\beta_{2}{\mathrm{Gfi}}_{it}\cdot I\left(T_{it}\geq \gamma \right)+\\ \rho {\mathrm{Control}}_{it}+\lambda_{i}+\eta_{t}+\varepsilon_{it} \end{array}$$

where the $T_{it}$ represents the threshold variables, including levels of carbon intensity, accessibility of medical services, standard of living for residents, and human capital levels. γ is the estimated threshold value. *I*( . ) is an indicator function which is assigned a value of 1 when the condition within the parentheses is satisfied, and a value of 0 otherwise. The definitions of other variables are the same as in Eq. (1).

### 3.2. Variable selection and data sources

#### 3.2.1. Explanatory variable

The level of green financial development (Gfi). According to the Guidance on Building a Green Financial System issued by China in 2016, and drawing on the study of Lee and Lee (2) and Huang et al. (20), four indicators of green credit, green securities, green insurance and green investment oriented to green development are used to construct an indicator system of green financial development at the provincial level in China through the entropy weight method. Among them, the level of green credit six high energy-consuming industries interest share to express. Because of the incomplete disclosure of information and inconsistent statistical caliber of China’s green credit data, and because there are only small differences in interest rates between industries, interest expenses can reflect the size of the loan (21, 22). Green securities are represented by the market capitalization of six high energy-consuming A-shares as a percentage of total A-share market capitalization, because the primary goal of green finance is to promote the green transformation of high energy-consuming and high-polluting industries. Green insurance includes a variety of products such as environmental pollution liability insurance, which is represented by the proportion of agricultural insurance scale in each province considering that environmental pollution liability insurance is still in its infancy in China (2, 20). Green investment is represented by the percentage of government investment in environmental pollution, because the government is the main investor in environmental protection in China, and the percentage of government investment in pollution control is a suitable indicator for green investment. The specific indicator selection and indicator definitions are shown in Table 1.

**Table 1:** Selection and definition of green finance indicators.

Tier 1 indicators	Secondary indicators	Indicator definition	Indicator properties
Level of green financial development	Green credit	Six high-energy-consuming industrial interest/industrial interest	−
	Green securities	Six high energy-consuming A-share market capitalization/total A-share market capitalization	−
	Green investment	Amount of investment in environmental treatment pollution/GDP	+
	Green insurance	Agricultural insurance income/total agricultural output	+

#### 3.2.2. Core explanatory variable

##### 3.2.2.1. Resident health level (health)

The usual method of gauging the health status of all residents in a certain area is by employing indices such as mortality rate, average life expectancy, and respondents’ subjective perceptions of their own health status (23). The “healthy China 2030” plan outlines the mortality rate as the principal health indicator, and thus this study uses the mortality rate of each region as a measurement of the resident health level (24).

#### 3.2.3. Threshold variables

##### 3.2.3.1. Carbon intensity (Cci)

Due to the lack of direct monitoring data on carbon emissions, this paper estimates the carbon emissions from 2007–2021 in 30 provinces in mainland China (excluding Tibet due to data availability) based on the reference method recommended by the IPCC, calculated as.


(3) $${\mathrm{CO}}_{2}=\overset{n}{\underset{i=1}{\sum }}E_{i}\times e_{i}\times p_{i}\times 44/12$$

(4) $$\mathrm{Cci}={\mathrm{CO}}_{2}/\mathrm{GDP}$$

where ${\mathrm{CO}}_{2}$ is the carbon emissions from fossil energy consumption in each province. $E_{i}$ represents the consumption of fossil energy of category *i*. $e_{i}\times p_{i}$ are the standard coal conversion factor and carbon emission factor of fossil energy of category *i*, respectively. *n* is the fossil energy type. 44/12 denotes ${\mathrm{CO}}_{2}$ the ratio of molecular weight to carbon (25). Cci is the carbon emission intensity of each province, and to eliminate the effects caused by price fluctuations, GDP is calculated using constant price GDP in 2000. The specific coefficients are listed in Table 2.

**Table 2:** Coefficients of standard coal and carbon emission used in calculating carbon emissions.

Energy type	Coal	Coke	Crude oil	Gasoline	Kerosene	Diesel	Fuel oil	Natural gas
Standard coal conversion factor	0.7143	0.9714	1.4286	1.4714	1.4714	1.4571	1.4286	1.33
Carbon emission factor	0.7559	0.855	0.5857	0.5538	0.5714	0.5921	0.6185	0.4483

Standard coal conversion factors are from Appendix 4 of the 2022 China Energy Statistics Yearbook; carbon emission factors are from IPCC (26, 27).

##### 3.2.3.2. Health care accessibility (Aci)

Firstly, the distribution of the number of health care institutions intuitively shows the popularity and spatial distribution of medical service facilities, so this paper uses the ratio of the number of health institutions to the year-end resident population to objectively quantify the distribution density of medical services (28). Second, the number of health personnel reflects the supply of human resources for medical services, which has a decisive influence on the quality and efficiency of medical services. For this reason, we chose the number of health technicians and the year-end resident population as indicators of human resource supply (29). Finally, the number of beds in health institutions demonstrates the carrying capacity of health care services, so this paper uses the ratio of the number of beds in health institutions to the year-end resident population to accurately assess the capacity of health care services (30). Therefore, this paper uses the ratio of the number of beds in health institutions to the year-end resident population to accurately assess the capacity of medical services. In summary, this paper evaluates the distribution, human resource provision, and service capacity of healthcare services through the utilization of the entropy weight method, building an index of healthcare service accessibility to assess the quantity and configuration of healthcare resources in different regions.

##### 3.2.3.3. Residents’ living standard (income)

Disposable income *per capita* is an important indicator to measure the living standard of residents in a region or a country, it reflects the level of income that residents can use for consumption and saving. High disposable income *per capita* usually means that residents have a higher quality of life, so this paper uses the logarithm of disposable income *per capita* of all residents to measure the living standard of residents in each region (31).

##### 3.2.3.4. Human capital level (Edu)

Elevated *per capita* years of schooling generally suggest an increased level of human capital, as more education typically results in advanced knowledge and skill sets. Therefore, this study employs the years of schooling *per capita* for individuals aged six and above in each province as a measure of the level human capital.

#### 3.2.4. Control variables

With reference to the existing literature, this paper selects control variables representing economic, environmental and social aspects of each region, including: economic development level (Tgdp): measured by the logarithm of the ratio of regional GDP to year-end resident population; green innovation level (patent): measured by the logarithm of the number of green inventions and utility model patents granted in each region; unemployment rate (Unem): measured by the registered unemployment rate of each region; social security level (Soc): measured by the logarithm of the amount of basic pension fund expenditures (32). The industrial structure (Stru): measured by the ratio of the value added of the tertiary industry to the regional GDP.

### 3.3. Data sources

This paper selects 30 provinces in China from 2007–2021 as the research sample. All data were obtained from the China Statistical Yearbook, China Health Statistical Yearbook, China Industrial Statistical Yearbook, China Insurance Statistical Yearbook, China Energy Statistical Yearbook, China Environmental Statistical Yearbook, China Science and Technology Statistical Yearbook and other provincial statistical yearbooks, as well as CEIC, CNRDS, CHOICE and Wind databases. And individual missing values are filled in by linear interpolation. The descriptive statistics of the main variables are shown in Table 3.

**Table 3:** Descriptive statistics of the main variables.

Variable	Explanation	Count	Mean	SD	Min	Max
Health	Residents’ health	450	0.779	0.182	0.220	0.889
Gfi	Green finance	450	0.759	0.181	0.198	1.427
Tgdp	Economic development level	450	4.612	0.250	3.894	5.265
Patent	Green innovation level	450	3.139	0.671	1.079	4.664
Unem	Unemployment rate	450	3.366	0.657	1.200	4.600
Soc	Social security level	450	6.740	0.457	5.377	7.603
Stru	Industry structure	450	0.476	0.093	0.298	0.837
Cci	Carbon intensity	450	4.205	3.539	0.440	19.91
Aci	Health care accessibility	450	3.082	0.557	0.228	3.818
Income	Residents’ living standard	450	4.255	0.239	3.683	4.892
Edu	Human capital level	450	9.075	0.975	6.785	12.68

## 4. Analysis of the empirical results

### 4.1. Baseline regression results

Before conducting the regression analysis, this paper uses a two-way fixed effects model to estimate the effect of regional green financial development level on residents’ health in China based on the Hausman test results. Stata 16 software was used for all analyses. The estimated results are shown in column (1) of Table 4, the coefficient of green finance (Gfi) is −0.0806 and passes the 5% significance level test, indicating that green financial development reduces regional mortality and has a significant effect on residents’ health there is a significant improvement effect on the health level of the residents, and the research hypothesis 1 holds.

**Table 4:** Baseline regression results.

Variable	(1)	(2)	(3)
	Baseline Regression	East	Midwest
Gfi	−0.0806^**^	−0.1115	−0.0812^**^
	(−2.73)	(−1.35)	(−2.54)
Tgdp	0.0444	0.0211	0.0494
	(0.35)	(0.08)	(0.33)
Patent	−0.1371^***^	−0.1749^*^	0.0118
	(−3.63)	(−2.15)	(0.41)
Unem	−0.0021	0.0153	0.0184
	(−0.20)	(0.82)	(0.99)
Soc	−0.0245	−0.1295	−0.0837
	(−0.39)	(−1.33)	(−1.13)
Stru	−0.2492^*^	−0.3793	0.0564
	(−1.74)	(−1.08)	(0.31)
_cons	1.0535	2.0078	0.8189
	(1.38)	(1.06)	(0.91)
Province FE	Yes	Yes	Yes
Year FE	Yes	Yes	Yes
*N*	450	165	165
*R* ^2^	0.557	0.398	0.703

*t*-values after considering clustering standard errors are in parentheses; ^***^, ^**^, ^*^ indicate significant correlations at the 1%, 5%, and 10% levels, respectively; same for the latter table.

In addition, significant differences exist in economic structure, environmental pressure, and healthcare standards between the eastern and central-western regions of China. These disparities may lead to heterogeneity in the impact of green finance on resident health levels across various regions. Therefore, to explore the diverse characteristics of green finance’s impact on resident health levels in different regions, this study segregates the national sample into two major sections—the east and the central-western. Separate regressions are then conducted within these regions to further evaluate the influence of green finance development on resident health levels. As shown in columns (2) and (3) of Table 4, the coefficient for green finance (Gfi) is not significant in the eastern region, but in the central-western region, the coefficient is −0.0812 and passes the 5% significance level test. The central-western region, in comparison to the eastern region, may have lower economic development, relatively poorer resource endowments, more pronounced environmental issues, and limited healthcare service quality and accessibility. This makes residents in the central-western regions more dependent on the potential benefits of green finance for environmental management and health improvement. Therefore, the impact of green finance on the health of residents in the central-western regions may be more significant than in the economically developed eastern regions with better healthcare conditions.

### 4.2. Endogeneity analysis

In order to avoid the bias caused by endogeneity issues, two instrumental variables are used in this paper to test the model using two-stage least squares method. First, the reciprocal of the distance between the province and the nearest port multiplied by the national green finance index of the year is applied as the instrumental variable (33, 34). This is because the concept of green finance in China is largely influenced by foreign influences (35). Some scholarly studies point out that elements such as foreign investment and trade play a key role in transmitting green financial awareness and development, and that green awareness is transmitted from coastal to inland areas (36, 37). In terms of geographic space, ports serve as the forefront of contact with other countries. It is shown that the further the distance from the port, the lower the level of green finance in the region will be. And this paper multiplies the distance with the time-varying green finance index to make the instrumental variable time-varying. In addition, this paper also selects green finance with a lag of 1 period as the instrumental variable for endogeneity testing (38). The estimation results are shown in Table 5 and the first stage *F*-values for the two instrumental variables are 33.21 and 130.77, respectively. The *F*-values are greater than 10 indicating that there is no weak instrumental variable problem. The second stage regression results show that the regression coefficient of the impact of green finance on residents’ health remains significantly negative, both with the explanatory variable lagged period as the instrumental variable and with the distance from each province to the nearest port as the instrumental variable, and the conclusion is consistent with hypothesis H1, which verifies the validity of the baseline regression model.

**Table 5:** Endogeneity analysis.

Variable	(1)		(2)	
	Distance		Gfi_1	
Gfi		−0.1030^**^		−0.1268^***^
		(−1.97)		(−3.75)
Distance	11.7674^***^			
	(5.76)			
Gfi_1			0.6381^***^	
			(11.44)	
Tgdp	0.1123	0.0446	0.1549	−0.0044
	(0.80)	(0.67)	(1.29)	(−0.06)
Patent	−0.0470	−0.1388^***^	−0.0219	−0.1255^***^
	(−0.93)	(−6.10)	(−0.66)	(−5.35)
Unem	0.0071	−0.0021	0.0008	−0.0030
	(0.52)	(−0.31)	(0.10)	(−0.45)
Soc	−0.2130^**^	−0.0285	−0.0947	−0.0417
	(−2.37)	(−0.79)	(−1.34)	(−1.09)
Stru	−0.3057	−0.2584^***^	−0.1048	−0.2838^***^
	(−2.37)	(−2.58)	(−0.60)	(−2.73)
Province FE	Yes	Yes	Yes	Yes
Year FE	Yes	Yes	Yes	Yes
Phase I *F*-value	33.21		130.77	
Cragg-Donald Wald *F* statistic		43.348		51.435
		{16.38}		{16.38}

### 4.3. Robustness test

To test the reliability of the benchmark regression results, this paper uses two methods for robustness testing: replacing the core explanatory variables and continuous variable tailing treatment. Originally, we continued to use the entropy weight method to construct the green finance index (Gfi1) by combining green fiscal policy with the above-mentioned green credit, green securities, green insurance and green investment into five indicators, which replaced the core explanatory variables of the benchmark regression model for the regression (34). One of the green fiscal indicators is measured by the ratio of fiscal environmental protection expenditure to fiscal general budget expenditure. In addition, due to the large sample size, all continuous variables in this paper are subjected to an upper and lower 1% tail shrinkage to mitigate the disturbance caused by outliers. The fundamental regression results are depicted in Table 6. The estimated coefficients, which quantify the influence of green financial development on residents’ health levels, stand at −0.0766 and −0.0772 respectively, significant at a 5% level. This further corroborates the robustness of the findings in our study.

**Table 6:** Robustness tests.

Variable	(1)	(2)
	Replacement of core explanatory variables	Shrinkage processing
Gfi1	−0.0766^**^	
	(−2.39)	
Gfi		−0.0772^**^
		(−2.57)
Tgdp	0.0261	0.0638
	(0.21)	(0.50)
Patent	−0.1337^***^	−0.1264^***^
	(−3.57)	(−3.48)
Unem	−0.0016	−0.0016
	(−0.15)	(−0.15)
Soc	−0.0409	−0.0010
	(−0.68)	(−0.02)
Stru	−0.2301	−0.2136
	(−1.66)	(−1.49)
_cons	1.2180	0.7810
	(1.61)	(1.08)
Province FE	Yes	Yes
Year FE	Yes	Yes
*N*	450	450
*R* ^2^	0.560	0.549

### 4.4. Threshold effect analysis

Green finance projects are characterized by long periodicity and high uncertainty, therefore, in the process of continuous response to the impact of green finance development on residents’ health, the threshold effect of the impact on residents’ health may be moderated by the external environment such as the level of local carbon intensity (Cci), the accessibility of medical services (Aci), the living standard of residents (Income) and the level of human capital (Edu), making the process shows certain stage characteristics. Therefore, this paper constructs a panel threshold model to further explore the nonlinear characteristics of the impact of green finance on residents’ health based on the different degrees of influence of the external environment in each region.

The results of the existence test in Table 7 show that the *F*-values in the single threshold test are 31.25, 37.01, 69.21 and 39.91, respectively, and the corresponding *p*-values are 0.0225, 0.0150, 0.0000 and 0.0100, respectively, indicating that there is a threshold effect in the model under the moderating effect of the four variables mentioned above. The *F*-values in the double threshold test are 20.21, 14.46, 6.51and 13.61, and the *p*-values are 0.1325, 0.1825, 0.7125 and 0.3650, respectively, so the double threshold effect is not considered to exist at the 0.05 significance level. Therefore, the single threshold effect model is chosen for the empirical analysis in this paper.

**Table 7:** Tests for the existence of threshold effects.

Threshold variables	Number of thresholds	*F*-value	*p*-value	BS number of times	Threshold		
					1%	5%	10%
Cci threshold	Single	31.25	0.0225	400	36.7362	26.9299	24.0219
	Double	20.21	0.1325	400	34.4004	25.8216	21.4686
Aci threshold	Single	37.01	0.0150	400	37.1740	27.5775	20.6590
	Double	14.46	0.1825	400	29.8502	21.2266	16.9068
Income threshold	Single	69.21	0.0000	400	36.2616	23.0642	19.8986
	Double	6.51	0.7125	400	27.5039	20.3126	17.2174
Edu Threshold	Single	39.91	0.0100	400	35.6702	27.2598	24.6737
	Double	13.61	0.3650	400	43.1798	27.6839	21.8990

In Table 8 the estimated value of carbon intensity (Cci) as the threshold variable is 3.6416, and its corresponding 95% confidence interval is (3.4605, 3.6428); the estimated value of accessibility to health care (Aci) as the threshold variable is 1.5977, and its corresponding 95% confidence interval is (1.5938, 1.6010), and the estimated value of living standard (income) as the threshold variable is estimated at 4.5516, and its corresponding 95% confidence interval is (4.5475, 4.5527), and the level of human capital (Edu) as the threshold variable is estimated at 10.7734, and its corresponding 95% confidence interval is (10.6091, 10.8156).

**Table 8:** Threshold estimation results and confidence intervals.

Threshold variables	Threshold	Estimated value	95% confidence interval
Cci threshold	First threshold value	3.6416^**^	(3.4605, 3.6428)
Aci threshold	First threshold value	1.5977^***^	(1.5938, 1.6010)
Income threshold	First threshold value	4.5516^***^	(4.5475, 4.5527)
Edu threshold	First threshold value	10.7734^**^	(10.6091, 10.8156)

**Figure 1:**
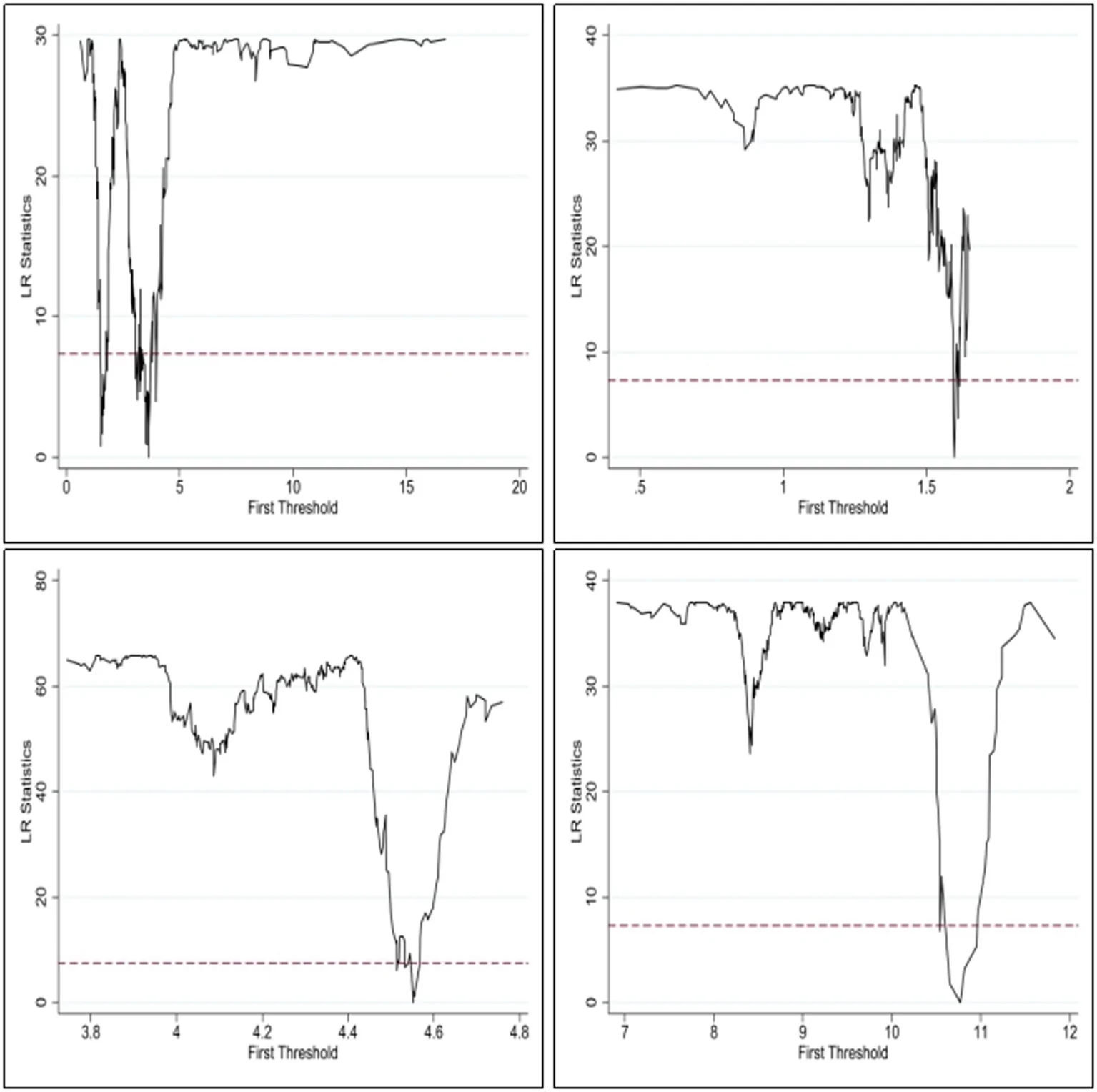
Graph of Cci, Aci, Income and Edu thresholds and likelihood ratio functions.

In the following, this paper performs consistency tests on the threshold estimates and the actual values. Based on the estimation results in Table 8, the likelihood ratio function is plotted in this paper. Among them, the horizontal axis of Figure 1 represents the threshold values of carbon intensity (Cci), health care accessibility (Aci), residents’ living standard (income) and human capital level (Edu) respectively, and the vertical axis all represent the likelihood ratio LR values. The dotted line represents the threshold value at the 95% confidence level. From Figure 1, it can be seen that the LR estimates corresponding to the four variables are significantly smaller than the threshold value of 7.35, so the above threshold estimates are true and valid.

In this paper, we use carbon intensity (Cci), accessibility of health care services (Aci), residents’ living standard (income) and human capital level (Edu) as threshold variables respectively, and the estimation results are shown in Table 9. In column (1), when the carbon intensity level is below the threshold value of 3.6416, the coefficient of green finance is not significant, while when the carbon intensity level crosses the threshold value of 3.6416, the coefficient of the effect of green finance on residents’ health is significantly −0.0933. This may be due to the fact that in areas with low carbon intensity, the environmental pressure is relatively low and therefore, the effect of green finance on residents’ health is insignificant. However, when the carbon intensity level exceeds the threshold value of 3.6416, the effect of green finance on the health of the population becomes significant and negative, i.e., green finance reduces the mortality rate in the regions. This may be due to the fact that in areas with high carbon intensity, where environmental pressure is higher, green finance investments and policies can be effective in reducing pollution and lowering carbon emissions, thus improving environmental conditions and the health of the population, and this result verifies hypothesis H2. In column (2), the coefficient of green finance is significantly −0.1054 at the 1% level when the accessibility of health care services is below the threshold value of 1.5977. When the accessibility of health care services crosses this threshold value, there is a non-linear characteristic of diminishing marginal effect of green finance on the health of the population. It indicates that when health care accessibility is low, green finance may have a greater impact on the health level of the population through its influence on economic and social activities. For example, green finance may direct the flow of funds to environmental industries, which may affect other industries, including the healthcare industry, thus affecting the supply of healthcare services and the health status of the population. And in areas with higher accessibility to healthcare services, the adequacy of healthcare resources may offset or diminish the impact of green finance, and this result verifies hypothesis H3.

**Table 9:** Threshold regression estimation results.

Variable	(1)	(2)	(3)	(4)
	Cci	Aci	Income	Edu
0.Gfi	−0.0468	−0.1054^***^	−0.0406	−0.0462
	(−1.60)	(−4.33)	(−1.25)	(−1.55)
1.Gfi	−0.0933^***^	−0.0471^*^	−0.1113^**^	−0.1258^***^
	(−3.38)	(−1.83)	(−2.64)	(−3.83)
Tgdp	0.0296	0.0673	−0.0967	−0.0135
	(0.25)	(0.57)	(−0.79)	(−0.11)
Patent	−0.125^***^	−0.1064^***^	−0.1439^***^	−0.1454^***^
	(−3.66)	(−3.15)	(−4.99)	(−4.30)
Unem	0.00131	−0.0023	0.0010	−0.0001
	(0.13)	(−0.22)	(0.10)	(−0.01)
Soc	0.00292	−0.0190	−0.0306	−0.0671
	(0.05)	(−0.36)	(−0.61)	(−1.20)
Stru	−0.274^*^	−0.1326	−0.3331^**^	−0.2557^*^
	(−2.02)	(−1.08)	(−2.50)	(−1.90)
_cons	0.912	0.8151	1.7074^**^	1.5585^**^
	(1.27)	(1.16)	(2.54)	(2.23)
Province FE	Yes	Yes	Yes	Yes
Year FE	Yes	Yes	Yes	Yes
*N*	450	450	450	450
*R* ^2^	0.582	0.591	0.617	0.592

In column (3), the coefficient of green finance is not significant when the residents’ living standard is below the threshold value of 4.5516, and when the residents’ living standard crosses the threshold value of 4.5516, the coefficient of green finance impact on residents’ health is significant at −0.1113. It indicates that at lower living standards, residents’ health is more influenced by basic survival needs and poverty status, and the impact of green finance may be diluted in this case (39). However, at higher living standards, green finance may have a positive impact on residents’ health by providing a cleaner environment and promoting healthy lifestyles, and this result supports hypothesis H4. The coefficient of green finance in column (4) is not significant when the level of human capital is below the threshold value of 10.7734, while the coefficient of the impact of green finance on the health of the population is significant at the 1% level when the level of human capital crosses this threshold value of −0.1258. It indicates that the management and service quality of financial institutions tend to be higher in areas rich in human capital, thus increasing the efficiency of capital investment in environmental and health industries, better improving environmental conditions, and thus promoting the health of the population (40). Also, since health is an expression of human capital (41), people with high human capital usually value health more and are willing to invest in it, a behavior that includes participation in green finance activities, which enhances their health (42), a result that supports hypothesis H5.

## 5. Conclusions and policy recommendations

This paper empirically examines the effects and mechanisms of regional green financial development on the residents’ health in China, using 30 Chinese provinces from 2007 to 2021. The results show that the development of green finance has a significant improvement effect on the health level of residents, and this conclusion still holds after considering the endogeneity and robustness issues. Moreover, the improvement effect of green finance on residents’ health is more obvious in the central and western regions, which indicates that residents in the central and western regions rely more on the health improvement effect brought by green finance for environmental management than those in the eastern regions with developed economy and relatively better resource endowment. In addition, the results of the threshold regression model indicate that under the single threshold constraint of carbon intensity, healthcare accessibility, residents’ living standard and human capital level in each region, the impact of green finance on residents’ health has a non-linear characteristic that is significantly influenced by external factors, specifically in provinces with higher carbon intensity level, residents’ living standard, human capital level and in provinces with lower healthcare accessibility, the effect of green finance on the promotion effect of green finance on residents’ health is more significant in provinces with lower access to medical services. The findings highlight the complexity of the role of green finance in influencing residents’ health, a dynamic that is largely influenced by regional socio-economic and environmental conditions. This provides new perspectives for understanding how green finance can be used to improve residents’ health, and these external factors, such as the socio-economic environment, should be fully taken into account in the implementation of green finance strategies in order to maximize the benefits of the impacts of green finance on improving residents’ health.

The policy implications of this paper are as follows: first, build a synergistic development ecosystem of green finance and public health. The transformation of traditional financial institutions into green finance should be promoted, and information-sharing among the financial, environmental and health sectors should be strengthened to break down the dilemma of “information silos.” According to specific socio-economic factors, when promoting green finance in regions with high carbon intensity, high living standards and human capital, but low access to health care services, consideration should be given to increasing investment in green projects and providing more environmentally friendly loans and preferential policies. This will optimize the allocation of green resources, stimulate the development of a green economy and further improve the health of the population. Second, improve financial literacy and enhance human capital levels. Policymakers should increase efforts to cultivate and attract high-level talents, and improve the financial literacy of the public by promoting financial education courses, conducting public lectures and seminars, or publishing financial knowledge through the media to ensure that the public has sufficient capacity to understand and utilize green financial products and services, thereby further enhancing the positive impact of green finance on the health of the population. Third, strengthen environmental and health policies. Since green finance is closely linked to the concept of environmental protection, policy makers should strengthen environmental protection measures such as carbon emission limits and waste management regulations, while promoting public health policies by strengthening disease prevention and popularizing public health education, thereby promoting complementary policy advantages and more effective use of green finance to improve environmental quality and enhance the health of the population, so as to achieve harmonious development of the population’s health and the economy and society.

### 5.1. Research limitations

Although this paper provides insight into the improvement effects of regional green finance development on the health of the population in China, there are still some aspects that need to be discussed in follow-up. For example, city-level data can be used to validate the results of this study in the future.

## Data availability statement

The original contributions presented in the study are included in the article/Supplementary material, further inquiries can be directed to the corresponding author.

## Author contributions

SF: conceptualization, methodology, software, data curation, and writing. FK: reviewing and editing. CL: reviewing and editing. All authors listed have made a substantial, direct, and intellectual contribution to the work and approved it for publication.

## Funding

This study was supported by the Shandong Provincial Health Economic Association 2023 Key Research Project (SDHEA2023-21) and the Shandong Provincial Adverse Drug Reaction Monitoring Center Project (2022SDADRKY04).

## Conflict of interest

The authors declare that the research was conducted in the absence of any commercial or financial relationships that could be construed as a potential conflict of interest.

## Correction note

A correction has been made to this article. Details can be found at: 10.3389/fpubh.2026.2013320.

## Publisher’s note

All claims expressed in this article are solely those of the authors and do not necessarily represent those of their affiliated organizations, or those of the publisher, the editors and the reviewers. Any product that may be evaluated in this article, or claim that may be made by its manufacturer, is not guaranteed or endorsed by the publisher.
