# Correction: Research on the impact of the development of green finance in the China region on residents' health

**DOI:** 10.3389/fpubh.2026.2013320

**Published:** 2026-10-22

**Authors:** Shuhao Fan, Fanchao Kong, Cheng Li

**Affiliations:** 1 Quality Management Department, Shandong Cancer Hospital and Institute, Shandong First Medical University and Shandong Academy of Medical Sciences, Jinan, China; 2 School of Statistics and Mathematics, Shandong University of Finance and Economics, Jinan, China

**Keywords:** green finance, residents' health, health care accessibility, carbon intensity, non-linear characteristics

The reference for [4] was erroneously written as “Dockery DW, Pope CA, Xu X, Spengler JD, Ware JH, Fay ME, et al. An association between air pollution and mortality in six US cities. N Engl J Med. (1993) 329:1753–9. doi: 10.1097/00043764-199502000-00008”. It should be “Dockery DW, Pope CA, Xu X, Spengler JD, Ware JH, Fay ME, et al. An association between air pollution and mortality in six US cities. *N Engl J Med*. (1993) 329:1753–9. doi: 10.1056/NEJM199312093292401”.

The reference for [6] was erroneously written as “Landrigan PJ, Fuller R, Acosta NJR, Adeyi O, Arnold R, Basu N, et al. The Lancet Commission on pollution and health. Lancet. (2018) 391:462–512. doi: 10.1016/S0140-6736(17)32345”. It should be “Landrigan PJ, Fuller R, Acosta NJR, Adeyi O, Arnold R, Basu N, et al. The Lancet Commission on pollution and health. *Lancet*. (2018) 391:462–512. doi: 10.1016/S0140-6736(17)32345-0”.

The reference for [8] was erroneously written as “Weber O, Feltmate B. Sustainable banking: managing the social and environmental impact of financial institutions. University of Toronto Press (2016)”. It should be “Weber O, Feltmate B. *Sustainable Banking: Managing the Social and Environmental Impact of Financial Institutions*. Toronto, ON: University of Toronto Press (2016). doi: 10.3138/9781442629325”.

The reference for [12] was erroneously written as “Bowler DE, Buyung-Ali LM, Healey JR, Jones JPG, Knight TM, Pullin AS. Does community forest management provide global environmental benefits and improve local welfare? Front Ecol Environ. (2012) 10:29–36. doi: 10.1890/110040”. It should be “Watts N, Adger WN, Agnolucci P, Blackstock J, Byass P, Cai W, et al. Health and climate change: policy responses to protect public health. *Lancet*. (2015) 386:1861–914. doi: 10.1016/S0140-6736(15)60854-6”.

The reference for [16] was erroneously written as “Neumayer E. Are left-wing party strength and corporatism good for the environment? Evidence from panel analysis of air pollution in OECD countries. Ecol Econ. (2003) 45:203–20. doi: 10.1016/S0921-8009(03)00012-0”. It should be “Franzen A, Vogl D. Two decades of measuring environmental attitudes: a comparative analysis of 33 countries. *Global Environ Chang*. (2013) 23:1001–8. doi: 10.1016/j.gloenvcha.2013.03.009”.

The reference for [17] was erroneously written as “Jacobsen GD. The Al Gore effect: an inconvenient truth and voluntary carbon offsets. J Environ Econ Manag. (2011) 61:67–78. doi: 10.1016/j.jeem.2010.08.002”. It should be “Gu Y, Ho KC, Yan C, Gozgor G. Public environmental concern, CEO turnover, and green investment: evidence from a quasi-natural experiment in China. *Energy Econ*. (2021) 100:105379. doi: 10.1016/j.eneco.2021.105379”.

The reference for [18] was erroneously written as “Demirguc-Kunt A, Klapper L, Singer D, Ansar S, Hess J. The global findex database 2017: measuring financial inclusion and the fintech revolution. World Bank Publications (2018)”. It should be “Anderson A, Robinson DT. Financial literacy in the age of green investment. *Rev Financ*. (2022) 26:1551–84. doi: 10.1093/rof/rfab031”.

The reference for [26] was erroneously written as “Energy Statistics Department of the National Bureau of Statistics of China. China energy statistical yearbook. China Statistics Press. (2022). 353–355”. It should be “Department of Energy Statistics, National Bureau of Statistics of China. *China Energy Statistical Yearbook 2022*. Beijing: China Statistics Press (2023). Appendix 4, p. 354–5. Available online at: https://www.zgtjcbs.com/quanbutushu/5506 (Accessed September 24, 2026)”.

The reference for [27] was erroneously written as “Paustian K, Ravindranath NH, Amstel AV. IPCC Guidelines for National Greenhouse Gas Inventories. International Panel on Climate Change. (2006). doi: 10.1016S1462-9011(99)00023-4”. It should be “IPCC. *2006 IPCC Guidelines for National Greenhouse Gas Inventories. Prepared by the National Greenhouse Gas Inventories Programme*. Eggleston HS, Buendia L, Miwa K, Ngara T, Tanabe K, editors. Hayama, Japan: Institute for Global Environmental Strategies (2006). Available online at: https://www.ipcc-nggip.iges.or.jp/public/2006gl (Accessed September 24, 2026)”.

The reference for [28] was erroneously written as “Shen F, Liu B, Luo F, Wu C, Chen H, Wei W. The effect of economic growth target constraints on green technology innovation. J Environ Manag. (2021) 292:112765. doi: 10.1016/J.JENVMAN.2021.112765”. It should be “Zhang T, Xu Y, Ren J, Sun L, Liu C. Inequality in the distribution of health resources and health services in China: hospitals versus primary care institutions. *Int J Equity Health*. (2017) 16:42. doi: 10.1186/s12939-017-0543-9”.

The reference for [39] was erroneously written as “Sulhi R. Inequality and the environmental Kuznets curve. Journal of Cleaner Production. (2019) 228(C). doi: 10.1016j.jclepro.2019.04.284”. It should be “Ridzuan S. Inequality and the environmental Kuznets curve. *J Clean Prod*. (2019) 228:1472–81. doi: 10.1016/j.jclepro.2019.04.284”.

The reference for [40] was erroneously written as “Becker GS. Investment in human capital: a theoretical analysis. J Polit Econ. (1962) 70:9–49. doi: 10.2307/1829103”. It should be “Becker GS. Investment in human capital: a theoretical analysis. *J Polit Econ*. (1962) 70:9–49. doi: 10.1086/258724”.

The reference for [41] was erroneously written as “Grossman M. On the concept of health capital and the demand for health. J Polit Econ. (1972) 80:223–55. doi: 10.2307/1830580”. It should be “Grossman M. On the concept of health capital and the demand for health. *J Polit Econ*. (1972) 80:223–55. doi: 10.1086/259880”.

The original version of this article has been updated.

